# Outcomes of Patients with Advanced Urothelial Carcinoma after Anti–programmed Death-(ligand) 1 Therapy by Fibroblast Growth Factor Receptor Gene Alteration Status: An Observational Study

**DOI:** 10.1016/j.euros.2022.11.001

**Published:** 2022-12-15

**Authors:** Arash Rezazadeh Kalebasty, David J. Benjamin, Yohann Loriot, Dimitrios Papantoniou, Arlene O. Siefker-Radtke, Andrea Necchi, Vahid Naini, Jenna Cody Carcione, Ademi Santiago-Walker, Spyros Triantos, Earle F. Burgess

**Affiliations:** a University of California Irvine, Irvine, CA, USA; b Institut Gustave Roussy, Université Paris‑Sud, Université Paris‑Saclay, Villejuif, France; c University of Texas MD Anderson Cancer Center, Houston, TX, USA; d Vita-Salute San Raffaele University, Department of Medical Oncology, IRCCS San Raffaele Hospital, Milan, Italy; e Janssen Research & Development, San Diego, CA, USA; f Janssen Research & Development, Raritan, NJ, USA; g Janssen Research & Development, Spring House, PA, USA; h Levine Cancer Institute, Atrium Health, Charlotte, NC, USA

**Keywords:** Anti–programmed death-(ligand) 1 therapy, Fibroblast growth factor receptor, *FGFR* alteration, Programmed death-(ligand) 1, Programmed cell death protein 1, Urothelial carcinoma

## Abstract

**Background:**

Clinical outcomes of anti–programmed death‑(ligand) 1 (anti–PD-[L]1) therapy in patients with locally advanced or metastatic urothelial carcinoma (mUC) and fibroblast growth factor receptor alterations (*FGFRa*+) remain unclear; recent studies have reported either comparable or poorer outcomes versus patients without *FGFR* alterations (*FGFRa*–)*.*

**Objective:**

To analyze the outcomes of patients with mUC and any *FGFRa* (mutations or fusions) who received anti–PD-(L)1 therapy.

**Design, setting, and participants:**

In this noninterventional, retrospective, multicenter study, clinical practice data were collected from *FGFRa+/*– patients who received prior immunotherapy between May 2018 and July 2019.

**Outcome measurements and statistical analysis:**

Investigator‑determined overall response rate (ORR), disease control rate (DCR), and overall survival (OS) were assessed in multivariate and unadjusted analyses.

**Results and limitations:**

Ninety-four patients (66% men; median age, 63 yr) with mUC and known *FGFR* status were included; 38 (40%) were *FGFRa+* and 56 (60%) were *FGFRa–*. In *FGFRa+* versus *FGFRa–* patients who received any line of anti–PD-(L)1 therapy (*n* = 92), ORR, DCR, and OS were 16% versus 26%, 29% versus 52% (relative risk: 1.14 [95% confidence interval {CI}, 0.92–1.40]; *p* = 0.3), and 8.57 versus 13.2 mo (hazard ratio [HR]: 1.33 [95% CI, 0.77–2.30]; *p* = 0.3), respectively. A multivariate analysis provided some evidence supporting shorter OS in *FGFRa*+ versus *FGFRa*– (any line of anti–PD-L[1] therapy; HR: 1.81 [95% CI, 0.99–3.31]; *p* = 0.054). Limitations include this study’s retrospective nature and a potential selection bias from small sample size.

**Conclusions:**

Some evidence of lower response rates and shortened OS following anti–PD-(L)1 therapy was observed in *FGFRa+* patients. The phase 3 THOR study (NCT03390504) will prospectively compare *FGFRa+* patients with advanced mUC treated with erdafitinib versus pembrolizumab.

**Patient summary:**

Patients with metastatic urothelial carcinoma and prespecified fibroblast growth factor receptor alterations (*FGFRa*) potentially have worse clinical outcomes when treated with anti–PD-(L)1 therapy than those without *FGFRa*.

## Take Home Message

This retrospective study found some evidence of potential lower response rates and shortened overall survival following anti–programmed death-(ligand) 1 therapy in patients with metastatic urothelial carcinoma with prespecified fibroblast growth factor receptor alterations (*FGFRa*) than in those without *FGFRa* who received prior immunotherapy between May 2018 and July 2019.

## 1 Introduction

In recent years, insights into the potential role of immunotherapies for bladder cancer have led to the approval of checkpoint inhibitors, such as atezolizumab (first-line treatment of platinum-ineligible patients regardless of programmed death ligand-1 [PD-L1] status and those with PD-L1+ [≥5%] tumors), avelumab (first-line maintenance irrespective of cisplatin eligibility), nivolumab (adjuvant treatment for those at a high risk of recurrence after radical resection and second-line treatment after platinum-based chemotherapy), and pembrolizumab (first-line treatment of platinum-ineligible patients or second-line treatment after platinum-based chemotherapy) for patients with locally advanced or metastatic urothelial carcinoma [1], [2], [3], [4]. While these immunotherapies have improved survival in patients with locally advanced or metastatic urothelial carcinoma [5], [6], [7], clinical benefit may vary depending on the molecular subtype and underlying immune landscape [8]. More specifically, response to checkpoint inhibitors may be dependent on T-cell infiltration of the tumor and T-cell function in the tumor microenvironment [8], [9], as improved outcomes have been observed in patients with programmed death-(ligand) 1 (PD-[L]1)-positive tumors [10]; however, as demonstrated in anti–PD-(L)1 clinical trials [6], [11], [12], [13], many patients with advanced urothelial carcinoma do not have PD-(L)1–positive tumors.

Fibroblast growth factor receptor (*FGFR*) alterations (*FGFRa*; mutations or fusions) are detected in approximately 15–20% of patients with locally advanced or metastatic urothelial carcinoma [14], [15]. Previous studies have shown that *FGFR3* mutations are encountered more frequently in luminal tumors, which are known to be comparatively less responsive to checkpoint inhibition, and that *FGFR3*-mutated bladder tumors are associated with decreased T-cell infiltration and low PD-L1 expression [15], [16], [17].

Several recent studies have reported the clinical outcomes of patients with *FGFRa* (*FGFRa+*) following anti–PD-(L)1 therapy, with differing outcomes [18], [19], [20], [21]. Only one of 22 patients enrolled in BLC2001 who had received prior immunotherapy was reported as having responded to immunotherapy, highlighting the need for additional treatment options [21]. First-line anti–PD-(L)1 treatment in patients with *FGFRa+* may be associated with poorer overall survival (OS); however, poorer OS was not observed in patients with *FGFRa+* treated with any-line or second-line anti–PD-(L)1 therapy [18]. Similarly, the JAVELIN Bladder 100 study reported poorer survival outcomes in patients with high versus low *FGFR3* gene expression who received first-line anti–PD-(L)1 therapy [20]. It was also shown that patients with *FGFRa+* who received anti–PD-(L)1 alone as first-line therapy had an adjusted risk of progression two times higher than that of patients with wild-type *FGFR* [22]. However, data from cohorts 1 and 2 of the IMVigor 210 study demonstrated no statistically significant difference in response rates in patients with mutant versus wild-type *FGFR3* with urothelial carcinoma treated with atezolizumab [19]. While patients from the PURE-01 study with high *FGFR3* gene expression showed a lower complete response rate versus those with low *FGFR3* gene expression following neoadjuvant pembrolizumab, the correlation between *FGFR3* activity or mutation/fusion and complete response was not established [23]. Real-world data from patients with advanced urothelial carcinoma treated with anti–PD-(L)1 therapy also demonstrated that *FGFR3*-altered and wild-type tumors have equivalent T-cell receptor diversity, with comparable objective response rates (ORRs), progression-free survival, and OS [24].

Recent data from cisplatin-ineligible patients with locally advanced or metastatic urothelial carcinoma showed that the majority of platinum-naïve patients who progressed to anti–PD-(L)1 therapy responded to enfortumab vedotin [25], [26]. Preliminary data from the NORSE study (NCT03473743) demonstrated improved efficacy with erdafitinib (a pan-FGFR inhibitor approved for the treatment of adult patients with locally advanced and metastatic urothelial carcinoma, and susceptible *FGFR3* or *FGFR2* genetic alterations, who have progressed during or following one or more prior lines of platinum-based chemotherapy) and the anti–PD-1 monoclonal antibody cetrelimab compared with erdafitinib alone (68% ORR [13/19] vs 33% ORR [6/18]) in patients with newly diagnosed locally advanced or metastatic urothelial carcinoma and *FGFRa* who were ineligible for cisplatin-based therapy, suggesting the potential value of combining therapies to overcome treatment resistance [27]. Therefore, treatment sequencing strategies should be considered carefully in light of emerging evidence on biomarker-directed therapies, including pan-FGFR inhibitors.

To build on this existing evidence, we conducted a retrospective analysis of the effects of any *FGFRa* in patients with locally advanced or metastatic urothelial carcinoma who received anti–PD-(L)1 therapy.

## 2 Patients and methods

### 2.1 Study design

**Fig. 1:**
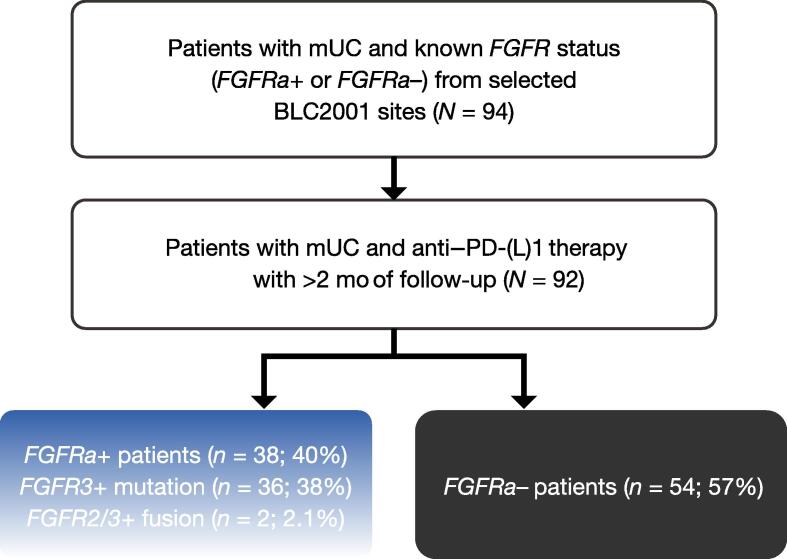
BLC0001 study design. Acceptable *FGFR* alterations included any *FGFR* mutation or gene fusion; copy number alterations/gene amplifications were not eligible in the absence of co-occurring *FGFR* mutations or fusions. FGFR = fibroblast growth factor receptor; *FGFRa*+/– = fibroblast growth factor receptor alteration positive/negative; mUC = metastatic urothelial carcinoma; PD-(L)1 = programmed death‑(ligand) 1.

This was a noninterventional, retrospective, multicenter study conducted at five sites in the USA and three sites in Europe (Fig. 1). Clinical practice data were collected from patients at selected BLC2001 study sites (NCT02365597) between May 2018 and July 2019 [21]. These patients were not enrolled in the BLC2001 study because of screening failure (either they did not meet the molecular eligibility criteria or they elected not to enroll in the trial), and were required to have previously been treated or treated subsequently with an anti–PD-(L)1 agent. Investigator‑determined ORR, investigator‑determined disease control rate (DCR), and OS per multivariate and unadjusted analyses were assessed for this study.

### 2.2 Study population

Eligible patients were diagnosed with urothelial carcinoma, received an anti–PD-(L)1 agent, and were either positive or negative for *FGFR* molecular alterations (any *FGFR* mutation or gene fusion, and copy number alterations/gene amplifications were not eligible; Supplementary Table 1). *FGFRa* status was tested at a central laboratory; RNA isolated from formalin-fixed, paraffin-embedded tumor samples was analyzed using a custom companion diagnostic reverse-transcriptase polymerase chain reaction assay (Qiagen, Hilden, Germany) at Almac Diagnostic Services, Craigavon, UK. This study was carried out prior to the approval of FGFR inhibitors (erdafitinib is the only FGFR inhibitor approved for the treatment of urothelial carcinoma). Prior treatment with erdafitinib was allowed before receiving anti–PD-(L)1 therapy, but only after the advanced diagnosis date. Treatment may have been with an anti–PD-(L)1 agent alone or in combination with chemotherapy or other treatments. Any number of prior lines of therapy was allowed, as was treatment with an anti–PD-(L)1 agent in either a clinical study or a treatment setting. Findings, data acquisition, and processing were conducted in accordance with the Declaration of Helsinki ethical standards, Good Clinical Practice guidelines, and all applicable local laws and regulations. When required by the study site, patients or their legally acceptable representatives provided written consent before participation. The study protocol and its amendments were approved by review boards at all participating institutions.

### 2.3 Statistical analysis

Estimated ORRs (with two-sided 95% Clopper-Pearson confidence intervals [CIs]) were calculated using normal approximation to the binomial distribution and presented by *FGFR* status (*FGFRa*+/–). ORR was defined as the proportion of patients with a best overall response of complete or partial response, as assessed by the investigator. DCR was defined as the proportion of patients with a best overall response of complete response, partial response, or stable disease, as assessed by the investigator. Results were provided for groupings of any-, first-, second-, and second- or higher-line immunotherapy. Relative risk was calculated to compare ORRs between patients who were *FGFR+* and *FGFR–*, and statistical significance was calculated using a chi-square test. OS analyses were conducted for any line of anti–PD-(L)1 therapy, first-line anti–PD-(L)1 therapy, second-line anti–PD-(L)1 therapy, and platinum-treated patients with a subsequent line of anti–PD-(L)1 therapy and presented by *FGFR* status. OS was measured from the start date of a specific line of therapy to the date of the patient’s death from any cause. For example, for an analysis involving first-line immunotherapy, OS was measured from the start date of first-line immunotherapy. Patients who terminated study participation or were lost to follow-up were censored at the date they were last known to be alive. Corresponding Kaplan–Meier survival function estimation and Cox proportional hazard models were implemented in the analysis of the data.

Subgroup analyses for OS were conducted for patients who received platinum-based therapy by *FGFR* status, that is, OS analysis for those who received any line of immunotherapy following platinum-based therapy and OS analysis for those who received immunotherapy immediately following platinum-based therapy. Bivariate and multivariate Cox regression models were performed using a selected set of potential prognostic variables and disease characteristic factors (sex, age, stage IV diagnosis, Bellmunt score, presence of transitional cell carcinoma, smoking status, and primary tumor location). Each factor was assessed individually in addition to the main factor of *FGFR* status in the bivariate model. Furthermore, factors were included as covariates in a multivariate model to assess their significance in the presence of other factors. Statistical analyses were performed using SAS version 9.4.

## 3 Results

### 3.1 Patient characteristics

Ninety-four patients with locally advanced or metastatic urothelial carcinoma and known *FGFR* status were included in this study. Of them, 38 (40%) were *FGFRa+* (36 [38%] had *FGFR3* mutations and two [2%] had *FGFR2/3* fusions) and 56 (60%) were *FGFRa–*. Demographics and baseline characteristics were balanced overall between *FGFRa+* and *FGFRa*– patients (Table 1). Patients had a median age (range) of 63 (41–85) yr, and 66% were men.

**Table 1:** Demographics, disease characteristics, and concomitant medications of patients who received any line of immunotherapy

Characteristics	*FGFRa+* (*n* = 38)^a^	*FGFRa–* (*n* = 54)
Age (yr), median (Q1, Q3)	63 (56.0, 69.0)	63 (55.0, 70.0)
Men, *n* (%)	28 (74)	33 (61)
Smoking history, *n* (%)		
Yes	26 (68)	34 (63)
Unknown	3 (8)	5 (9)
Hemoglobin level (g/dl), *n* (%)		
<10	8 (21)	10 (19)
≥10	24 (63)	39 (72)
Unknown	6 (16)	5 (9)
ECOG PS, *n* (%)		
0	14 (37)	20 (37)
1	13 (34)	24 (44)
2	3 (7.9)	6 (11)
Unknown	8 (21)	4 (7.4)
Bellmunt score, *n* (%)		
0	14 (37)	20 (37)
1	14 (37)	25 (46)
2	5 (13)	8 (15)
Unknown	5 (13)	1 (1.9)
Primary tumor location, *n* (%)		
Bladder	26 (68)	41 (76)
Urethra	1 (2.6)	0
Ureter/renal pelvis	11 (29)	12 (22)
Unknown	0	1 (1.9)
Histology type, *n* (%)		
Urothelial carcinoma	32 (84)	44 (82)
Urothelial carcinoma with variant histology	5 (13)	8 (15)
Unknown/not documented	1 (2.6)	2 (3.7)
Prior neoadjuvant/adjuvant chemotherapy^b^, *n* (%)		
Yes	1 (2.6)	2 (3.7)
Number of patients taking any immunotherapy after diagnosis, *n* (%)		
First line	14 (37)	10 (19)
Second line or higher^c^	25 (66)	38 (70)
Second line	11 (29)	25 (46)
Third line or higher	16 (66)	16 (30)
Prior treatments^d^, *n* (%)		
Patients receiving immunotherapy-containing regimens	38 (100)	54 (100)
Monotherapy	21 (55)	37 (69)
Combination immunotherapy	7 (18)	3 (5.6)
Immunotherapy-chemotherapy combination	4 (11)	3 (5.6)
Patients receiving chemotherapy-containing regimens	36 (95)	47 (87)
Monotherapy	6 (17)	21 (45)
Chemotherapy-chemotherapy combination	30 (83)	45 (96)
Immunotherapy-chemotherapy combination	4 (11)	3 (6.4)

ECOG PS = Eastern Cooperative Oncology Group performance status; FGFR = fibroblast growth factor receptor; *FGFRa+/–* = fibroblast growth factor receptor alteration positive/negative; PD-(L)1 = programmed death‑(ligand) 1.

a Nine *FGFRa+* patients received treatment with FGFR inhibitors, but none of these patients received this treatment before receiving anti–PD-(L)1 therapy after the advanced diagnosis date.

b Before advanced diagnosis date, defined as the date of first diagnosis of urothelial carcinoma (when available) or the date of first diagnosis of metastatic disease.

c Includes patients who received multiple lines of immunotherapy.

d The same patient may be counted as having received immunotherapy and chemotherapy. No patients had prior anti–PD-(L)1 monotherapy before the advanced diagnosis date.

All patients in the *FGFRa+* cohort and 54 patients in the *FGFRa–* cohort received anti–PD-(L)1 therapy (Fig. 1); two patients were excluded for not meeting the study eligibility criteria (one received an anti–PD-[L]1 agent prior to the date of advanced urothelial carcinoma diagnosis and a second did not receive an anti–PD-[L]1 agent). After the advanced diagnosis date, nine patients received FGFR inhibitor treatment before receiving anti–PD-(L)1 therapy, and most patients (63%) had received anti‑PD‑(L)1 monotherapy; the most common agent was atezolizumab (Table 1). The proportion of patients receiving an immunotherapy/immunotherapy combination was higher in the *FGFRa+* group than in the *FGFRa−* group (18% vs 6%).

### 3.2 Outcomes by FGFR status

The median follow-up duration was 31.1 (range, 5.7–299.9) mo. There was some evidence of lower ORRs and DCRs to anti–PD-(L)1 therapy in *FGFRa+* versus *FGFRa*– patients regardless of the number of prior lines of therapy; however, the difference in rates between groups did not reach conventional levels of statistical significance (Table 2). Among the 92 patients who received any line of anti–PD-(L)1 therapy, ORRs in those with *FGFRa+* and *FGFRa–* were 16% and 26%, respectively (relative risk: 1.14 [95% CI, 0.92–1.40]; *p* = 0.3).

**Table 2:** Best overall response and overall survival by *FGFR* status and the sequence number in which prior immunotherapy was used

	*FGFRa+*	*FGFRa–*	Total
Any line of anti–PD-(L)1 therapy, *n*	38	54	92
ORR, % (95% CI)	16 (4.2–27)	26 (14–38)	22 (13–30)
RR (95% CI), *p* value^a^	1.14 (0.92–1.40), 0.3		–
DCR, % (95% CI)	29 (15-43)	52 (39–65)	42 (32–53)
OS (mo), median (95% CI)	8.57 (6.05–18.3)	13.2 (7.29–39.2)	11.4 (7.69–19.7)
HR (95% CI), *p* value	1.33 (0.77–2.30), 0.3		–
First line of anti–PD-(L)1 therapy, *n*	14	10	24
ORR, % (95% CI)	29 (4.9–52)	30 (1.6–58)	29 (11–47)
RR (95% CI), *p* value^a^	1.02 (0.60–1.72), >0.9		–
DCR, % (95% CI)	36 (11–61)	60 (30–90)	46 (26–66)
OS (mo), median (95% CI)	18.3 (5.88–NE)	25.3 (2.46–25.3)	18.3 (7.29–25.3)
HR (95% CI), *p* value	1.12 (0.33–3.84), 0.9		–
Second line of anti–PD-(L)1 therapy, *n*	11	25	36
ORR, % (95% CI)	9.1 (0–26)	20 (4.3–36)	17 (4.5-29)
RR (95% CI), *p* value^a^	1.14 (0.87–1.49), 0.4		–
DCR, % (95% CI)	18 (0–41)	56 (37–76)	44 (28–61)
OS (mo), median (95% CI)	7.69 (2.96–19.7)	11.0 (5.36–39.2)	11.0 (5.36–22.0)
HR (95% CI), *p* value	1.47 (0.60–3.60), 0.4		–
Second or higher line of anti–PD-(L)1 therapy, *n*	25	38	63
ORR, % (95% CI)	8.0 (0–19)	21 (8.1–34)	16 (6.8–25)
RR (95% CI), *p* value^a^	1.17 (0.95–1.42), 0.2		–
DCR, % (95% CI)	24 (7.3–41)	50 (34–66)	40 (28–52)
Platinum-treated patients with subsequent any line of anti–PD-(L)1 therapy, *n*	25	40	65
ORR, % (95% CI)	12 (0–25)	25 (12–38)	20 (10–30)
RR (95% CI), *p* value^a^	1.17 (0.93–1.48), 0.2		–
DCR, % (95% CI)	28 (10–46)	53 (37–68)	43 (31–55)
OS (mo), median (95% CI)	7.52 (5.52–19.7)	11.4 (5.36–22.0)	10.3 (7.06–15.7)
HR (95% CI), *p* value	1.24 (0.66–2.33), 0.5		–

Anti–PD-(L)1 = anti–programmed death-(ligand)1; CI = confidence interval; DCR = disease control rate; FGFR = fibroblast growth factor receptor; *FGFRa+/–* = fibroblast growth factor receptor alteration positive/negative; HR = hazard ratio; NE = not evaluable; ORR = objective response rate; OS = overall survival; RR = relative risk.

a *p* values were calculated using a chi-square test.

**Fig. 2:**
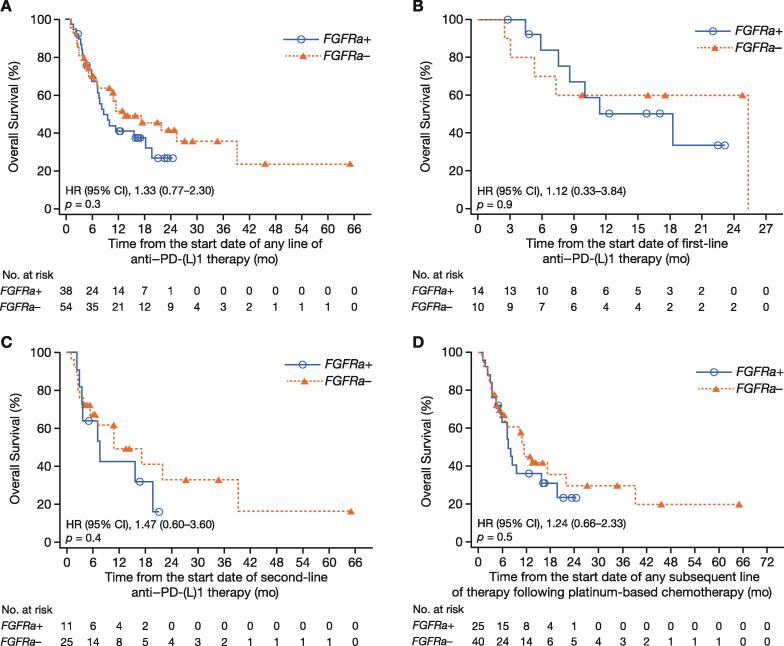
Kaplan–Meier curves of overall survival. Overall survival measured to the date of the patient’s death from any cause from the date of starting: (A) any line of anti–PD-(L)1 therapy, (B) first line of anti–PD-(L)1 therapy, (C) second line of anti–PD-(L)1 therapy, and (D) any subsequent line of anti–PD-(L)1 therapy following platinum-based chemotherapy. Patients who terminated study participation or were lost to follow-up were censored at the date they were last known to be alive. CI = confidence interval; *FGFRa+/–* = fibroblast growth factor receptor alteration positive/negative; HR = hazard ratio; PD-(L)1 = programmed death‑(ligand) 1.

Although some evidence of shorter median OS was also observed in the univariate analysis for patients with *FGFRa+* versus those with *FGFRa–*, irrespective of the sequence number in which prior immunotherapy was used, the difference in OS between groups did not reach conventional levels of statistical significance (Table 2 and Fig. 2). The median OS (from diagnosis or from first treatment with first-line therapy) in patients with *FGFRa+* and *FGFRa–* treated with any line of anti–PD-(L)1 was 8.57 and 13.2 mo (hazard ratio [HR]: 1.33 [95% CI, 0.77–2.30]; *p* = 0.3), respectively.

Among the 24 patients who received first-line immunotherapy, the median OS was 18.3 mo in those who were *FGFRa+* (*n* = 14) and 25.3 mo in those who were *FGFRa–* (*n* = 10; HR: 1.12 [95% CI, 0.33–3.84]; *p* = 0.9). Among the 36 patients who received second-line immunotherapy treatment, the median OS was 7.69 mo in those who were *FGFRa+* (*n* = 11) and 11.0 mo in those who were *FGFRa–* (*n* = 25; HR: 1.47 [95% CI, 0.60–3.60]; *p* = 0.4).

**Fig. 3:**
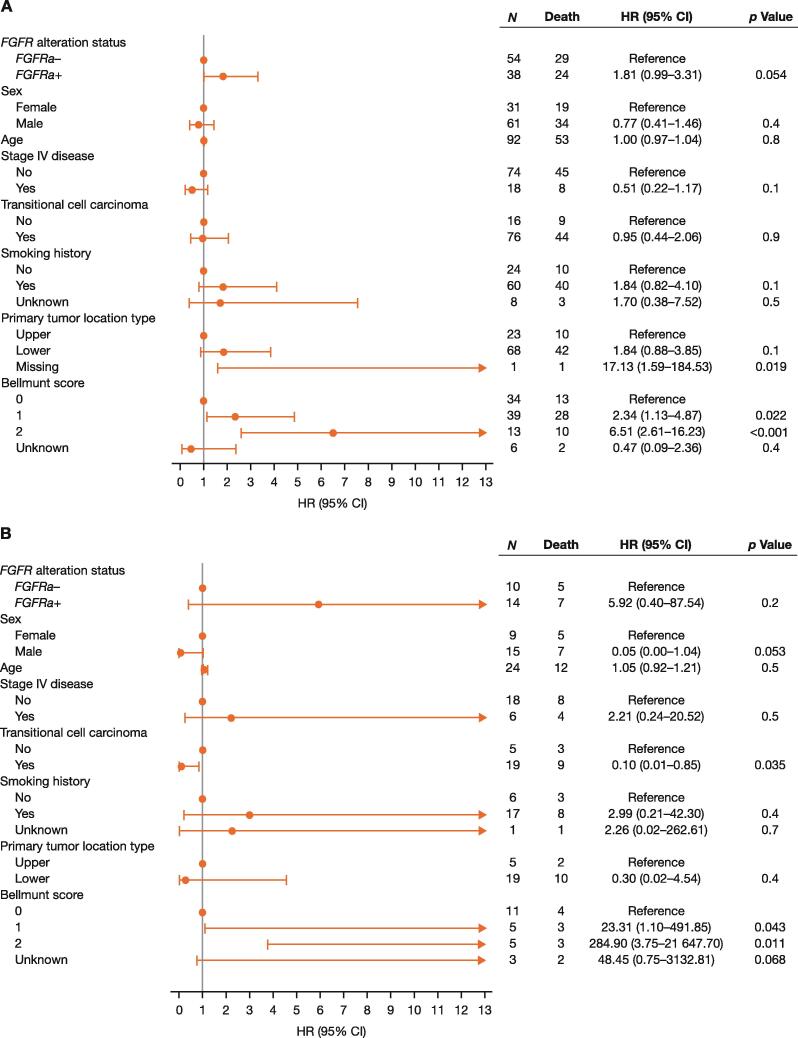

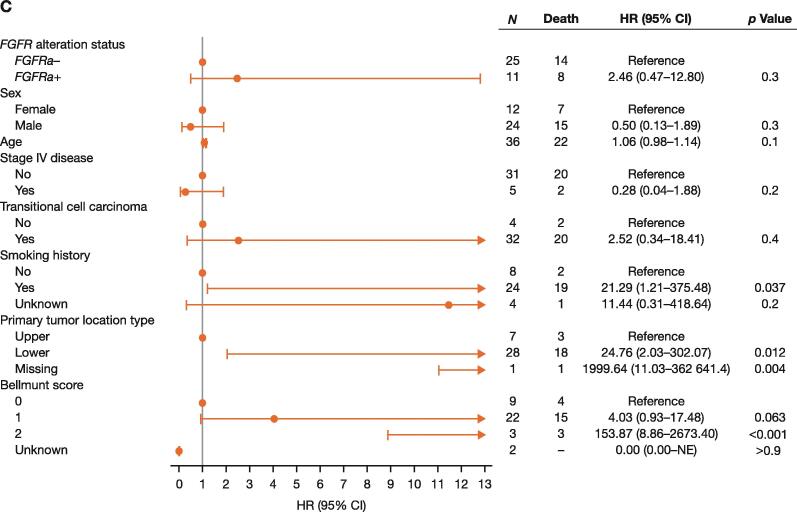
Multivariate analysis of overall survival in patients treated with (A) any line of prior anti–PD-(L)1 therapy, (B) first-line anti–PD-(L)1 therapy, and (C) second-line anti–PD-(L)1 therapy. CI = confidence interval; *FGFRa+/–* = fibroblast growth factor receptor alteration positive/negative; HR = hazard ratio; NE = not evaluable; PD-(L)1 = programmed death‑(ligand) 1; PH = proportional hazard.

OS was shorter in *FGFRa+* patients than in *FGFRa–* patients who received prior platinum chemotherapy and subsequent anti–PD-(L)1 therapy; however, the difference was not statistically significant (Table 2 and Fig. 2). A multivariate analysis provided some evidence for shorter OS in *FGFRa+* than in *FGFRa–* patients, with an HR of 1.81 (95% CI, 0.99–3.31) in those who had any line of anti–PD-(L)1 therapy (*p* = 0.054), 5.92 (95% CI, 0.40–87.54) in those who received first-line anti–PD-(L)1 treatment (*p* = 0.2), and 2.46 (95% CI, 0.47–12.80) in those who had second-line anti–PD-(L)1 therapy (*p* = 0.3); however, the difference in OS between groups did not reach conventional levels of statistical significance (Fig. 3).

## 4 Discussion

In this retrospective analysis of patients with locally advanced/metastatic urothelial carcinoma, some evidence of poorer outcomes was observed in those with *FGFR*+ alterations following anti–PD-(L)1 therapy, highlighting the potential unmet need in this patient group. Irrespective of the prior line of anti–PD-(L)1 therapy, there was some evidence toward lower ORRs and DCRs in *FGFRa+* than in *FGFR−* patients. Similarly, there was some evidence of shorter OS in the *FGFRa+* cohort than in the *FGFRa–* cohort. The median OS of 10.97 mo for patients with advanced urothelial carcinoma following second-line anti–PD-(L)1 treatment was similar to that reported in studies of second-line anti–PD-(L)1 therapy (eg, 10.3 mo for pembrolizumab [28], 8.7 mo for nivolumab [29], and 11.1 mo for atezolizumab) [6]. It is worth noting that this study was carried out prior to the approval of FGFR inhibitors for any indication.

Importantly, recent promising data on the use of enfortumab vedotin in cisplatin-ineligible patients with locally advanced or metastatic urothelial carcinoma who progressed after anti–PD-(L)1 therapy [25], [26] suggest that appropriate treatment sequencing strategies should be considered as clinical evidence with biomarker-directed therapies, including FGFR inhibitors, continues to emerge. Other clinical studies evaluating FGFR inhibition in patients with advanced urothelial carcinoma whose tumors expressed *FGFRa* also found a poor response to prior immunotherapy. While it may not be surprising to see a lower response rate to anti–PD-(L)1 in a relapsed/refractory population, it is interesting that 59% of patients in the BLC2001 primary analysis responded to erdafitinib following anti–PD-(L)1 therapy [21]. Likewise, in a phase 1 study of rogaratinib in patients with advanced cancers selected according to *FGFR* mRNA expression, approximately 30% of patients with urothelial carcinoma who received prior immunotherapy responded to rogaratinib [30]. However, these results are not conclusive since it was also demonstrated that *FGFR3* alterations do not preclude a response to nivolumab in metastatic urothelial cancer [31], suggesting that further studies are needed in this setting to clarify the potential effects of *FGFRa* on clinical outcomes.

The current study was limited by its retrospective nature, the relatively small number of patients, potential selection bias, and nonstatistically significant results. Patients were selected for their suitability to receive an FGFR inhibitor; *FGFRa–* patients who were included in this analysis failed screening for the BLC2001 study because they did not meet the molecular eligibility criteria. Likewise, *FGFRa*+ patients who were not enrolled in the BLC2001 study because of screening failure (or elected not to enroll in the trial) may not be representative of *FGFRa*+ patients. Therefore, patients included in this analysis do not represent a randomly selected population, which is a limitation of this study. However, the baseline data from the two cohorts (*FGFRa+* vs *FGFRa–* patients) were generally similar and prognostically comparable (based on Bellmunt scores), supporting the assessment of anti–PD-(L)1 therapy outcomes between these groups. Another potential source of selection bias is that, owing to small numbers of patients in each cohort, patients who were permitted to receive an anti–PD-(L)1 agent alone or in combination with chemotherapy or other treatments, any number of prior lines of therapy, and treatment with an anti–PD-(L)1 agent in either a clinical study or treatment setting were pooled together. Furthermore, patients with copy number alterations and gene amplifications were not considered, as this study was designed to investigate mutations and fusions that were more reflective of the population that are clinically targeted by FGFR inhibitors. In addition, it was not possible to ascertain the dynamics of *FGFRa* positivity throughout patients’ treatment course, highlighting the potential value for using circulating tumor DNA testing to monitor genomic alterations over time, as an alternative to tumor tissue testing [32]. Of the 38 *FGFRa*+ patients, nine received FGFR inhibition prior to receiving immunotherapy; this additional targeted treatment for *FGFRa*+ patients represents a source of a potential bias as one could expect different outcomes from these patients. However, the evidence toward worse outcomes in *FGFRa*+ patients despite this additional treatment shows a clinical need in this patient population.

The findings of this study contribute to the emerging data on the predictive value of *FGFRa* on outcomes of patients with advanced or metastatic urothelial carcinoma following anti–PD-(L)1 therapy and the unmet medical need in this targetable patient population. Further studies are needed to confirm these results in a larger patient cohort and to clarify whether other underlying concomitant genomic alterations dictate the treatment response.

## 5 Conclusions

In this retrospective study, there was some evidence of lower ORRs and DCRs in patients with *FGFRa+* versus those with *FGFRa–* and advanced or metastatic urothelial carcinoma who had received anti–PD-(L)1 therapy. A multivariate analysis showed some evidence toward shorter median OS in patients with *FGFRa+* versus those with *FGFRa–* in this cohort of patients treated with immunotherapy. These data provide some evidence toward the hypothesis that patients with *FGFR* gene alterations have poor outcomes with anti–PD-(L)1 agents and contribute to the emerging data on outcomes of *FGFRa+* patients with available therapies.

This work was previously (virtual) presented at the European Society for Medical Oncology Congress, September 19–21, 2020 (abstract 757P).

  ***Author contributions*:** Arash Rezazadeh Kalebasty had full access to all the data in the study and takes responsibility for the integrity of the data and the accuracy of the data analysis.

*Study concept and design*: Rezazadeh Kalebasty, Loriot, Santiago-Walker, Siefker-Radtke.

*Acquisition of data*: Rezazadeh Kalebasty, Papantoniou, Siefker-Radtke, Necchi, Burgess.

*Analysis and interpretation of data*: Rezazadeh Kalebasty, Benjamin, Siefker-Radtke, Santiago-Walker, Carcione, Burgess.

*Medical review of data*: Rezazadeh Kalebasty, Naini, Burgess.

*Drafting of the manuscript*: Rezazadeh Kalebasty, Benjamin, Papantoniou, Siefker-Radtke, Necchi, Carcione, Santiago-Walker.

*Critical revision of the manuscript for important intellectual content*: All listed authors.

*Statistical analysis*: Carcione.

*Obtaining funding*: Naini.

*Administrative, technical, or material support*: Naini.

*Supervision*: Rezazadeh Kalebasty, Siefker-Radtke.

*Other*: None.

  ***Financial disclosures:*** Arash Rezazadeh Kalebasty certifies that all conflicts of interest, including specific financial interests and relationships and affiliations relevant to the subject matter or materials discussed in the manuscript (eg, employment/affiliation, grants or funding, consultancies, honoraria, stock ownership or options, expert testimony, royalties, or patents filed, received, or pending), are the following: Arash Rezazadeh Kalebasty holds stocks in ECOM Medical; has had an advisory role for AstraZeneca, Bayer, Bristol Myers Squibb, EMD Serono, Exelixis, Immunomedics, Genentech, Gilead Sciences, Novartis, and Pfizer; has received speaker’s fees from Amgen, Astellas Medivation, AVEO, AstraZeneca, Bristol Myers Squibb, Eisai, EMD Serono, Exelixis, Genentech/Roche, Gilead Sciences, Janssen, Merck, Myovant Sciences, Novartis, Pfizer, Sanofi, and Seattle Genetics/Astellas; received research funding from Astellas Pharma, AstraZeneca, Bavarian Nordic, Bayer, BeyondSpring, BioClin Therapeutics, Bristol Myers Squibb, Clovis Oncology, Eisai, Epizy, Exelixis, Genentech, Immunomedics, Janssen, Macrogenics, and Seattle Genetics; and has received travel fees from Astellas Medivation, AstraZeneca, Bayer, Eisai, Exelixis, Genentech, Janssen, Novartis, Pfizer, and Prometheus Laboratories. David Benjamin declares no conflict of interests. Yohann Loriot has received consulting fees from Janssen, Astellas Pharma, Roche, AstraZeneca, MSD Oncology, Clovis Oncology, Seattle Genetics, and Bristol Myers Squibb; and reports travel and reimbursement for accommodations or expenses from Astellas Pharma, Janssen Oncology, Roche, AstraZeneca, MSD Oncology, Clovis Oncology, Seattle Genetics, and Bristol Myers Squibb, all outside the submitted work. Dimitrios Papantoniou reports consulting fees from MSD and speaker’s fees from Ipsen, all outside the submitted work. Arlene O. Siefker-Radtke received support from NIH, Michael and Sherry Sutton Fund for Urothelial Cancer, Janssen, Takeda, Bristol Myers Squibb, BioClin Therapeutics, Nektar, Merck Sharp & Dohme, and Basilea; has received consulting fees from Janssen, Merck, NCCN, Bristol Myers Squibb, AstraZeneca, Bavarian Nordic, Ideeya, Loxo, Immunomedics, Merck Sharp & Dohme, Seattle Genetics, Nektar, Genentech, EMD Serono, Mirati Therapeutics, and Basilea; and has patents planned, issued, or pending related to molecular testing in muscle-invasive bladder cancer, all outside the submitted work. Andrea Necchi received personal fees from Bayer during the conduct of the study; received consulting fees from Merck Sharp & Dohme, Roche, Bayer, AstraZeneca, Clovis Oncology, Janssen, Seattle Genetics/Astellas, Bristol Myers Squibb, GlaxoSmithKline, and Ferring; received honoraria from Roche, Merck, AstraZeneca, Janssen, Foundation Medicine, and Bristol Myers Squibb; received support for attending meetings and/or travel from Roche, Merck Sharp & Dohme, AstraZeneca, Janssen, and Rainier Therapeutics; has stock or stock options for an immediate family member from Bayer; and received other financial or nonfinancial interests from Merck Sharp & Dohme, AstraZeneca, and Ipsen, all outside the submitted work. Earle F. Burgess has received grants or contracts from Pfizer and Astellas Pharma; received speaker's fees from Exelixis and AstraZeneca; received consulting fees from Merck Sharp & Dohme, Janssen, Pfizer, Novartis; and has stock or stock options from Exelixis, Becton Dickinson, Gilead Sciences, Medtronic, Arvinas, and Macrogenics, all outside the submitted work. Vahid Naini, Jenna Cody Carcione, Ademi Santiago-Walker, and Spyros Triantos are employees of Janssen Pharmaceuticals.

  ***Funding/support and role of sponsor*:** This study was funded by Janssen Research & Development. The sponsor was involved in the design and conduct of the study; analysis and interpretation of the data; preparation, review, and approval of the manuscript; and decision to submit the manuscript for publication. Funding for editorial assistance was provided by Janssen Global Services, LLC.

  ***Data sharing*:** Janssen Pharmaceutical Companies of Johnson & Johnson’s data sharing policy is available at https://www.janssen.com/clinical-trials/transparency. As noted on this site, requests for study data access can be submitted through Yale Open Data Access (YODA) Project site at http://yoda.yale.edu.

  ***Acknowledgments*:** Writing assistance was provided by Khalida Rizi, PhD, of Parexel. Erdafitinib (JNJ-42756493) was discovered in collaboration with Astex Pharmaceuticals.
