# Supplementary material for: Outcomes of Patients with Advanced Urothelial Carcinoma after Anti–programmed Death-(ligand) 1 Therapy by Fibroblast Growth Factor Receptor Gene Alteration Status: An Observational Study
Source: Eur Urol Open Sci. 2022 Dec 15;47:48–57. doi: 10.1016/j.euros.2022.11.001 (PMC9806713; doi:10.1016/j.euros.2022.11.001)
Supplement: Supplementary data 1 [file mmc1.docx]

**SUPPLEMENTARY MATERIAL**

**Outcomes of Patients with Advanced Urothelial Carcinoma after Anti–Programmed Death-(Ligand) 1 Therapy by Fibroblast Growth Factor Receptor Gene Alteration Status: An Observational Study**

Arash Rezazadeh Kalebasty,^a^ David J. Benjamin,^a^ Yohann Loriot,^b^ Dimitrios Papantoniou,^b^ Arlene O. Siefker-Radtke,^c^ Andrea Necchi,^d^ Vahid Naini,^e^ Jenna Cody Carcione,^f^ Ademi Santiago-Walker,^g^ Spyros Triantos,^g^ Earle F. Burgess^h^

^a^University of California Irvine, Irvine, CA, USA; ^b^Institut Gustave Roussy, Université Paris‑Sud, Université Paris‑Saclay, Villejuif, France; ^c^University of Texas MD Anderson Cancer Center, Houston, TX, USA; ^d^Vita-Salute San Raffaele University, Department of Medical Oncology, IRCCS San Raffaele Hospital, Milan, Italy; ^e^Janssen Research & Development, San Diego, CA, USA; ^f^Janssen Research & Development, Raritan, NJ, USA; ^g^Janssen Research & Development, Spring House, PA, USA; ^h^Levine Cancer Institute, Atrium Health, Charlotte, NC, USA

CONTENTS

**Supplementary Table 1.** List of fibroblast growth factor alterations.

**Supplementary Table 1.** List of *FGFR* alterations.

| *FGFR* alteration | Total (n=38) |
| --- | --- |
| *FGFR*2 fusion | 1 |
| *FGFR*3 fusion | 1 |
| *FGFR*3 mutation | 36 |

*FGFR* = fibroblast growth factor receptor.
